# Sex Differences in Cardiac Mitochondria in the New Zealand Obese Mouse

**DOI:** 10.3389/fendo.2018.00732

**Published:** 2018-12-04

**Authors:** Cathleen John, Jana Grune, Christiane Ott, Kerstin Nowotny, Stefanie Deubel, Arne Kühne, Carola Schubert, Ulrich Kintscher, Vera Regitz-Zagrosek, Tilman Grune

**Affiliations:** ^1^Department of Molecular Toxicology, German Institute of Human Nutrition Potsdam-Rehbruecke, Potsdam, Germany; ^2^German Center for Cardiovascular Research (DZHK), Partner Site Berlin, Berlin, Germany; ^3^Institute of Physiology, Charité Universitätsmedizin Berlin, Berlin, Germany; ^4^Institute of Pharmacology, Center for Cardiovascular Research, Charité -Universitätsmedizin Berlin, Berlin, Germany; ^5^Center for Cardiovascular Research, Charité Universitätsmedizin Berlin, Berlin, Germany; ^6^Institute for Gender in Medicine, Charité Universitätsmedizin Berlin, Berlin, Germany; ^7^Institute of Nutritional Science, University of Potsdam, Potsdam, Germany; ^8^German Center for Diabetes Research, Oberschleißheim, Germany

**Keywords:** NZO, heart, obesity, mitochondrial function, echocardiography, systolic function

## Abstract

**Background:** Obesity is a risk factor for diseases including type 2 diabetes mellitus (T2DM) and cardiovascular disorders. Diabetes itself contributes to cardiac damage. Thus, studying cardiovascular events and establishing therapeutic intervention in the period of type T2DM onset and manifestation are of highest importance. Mitochondrial dysfunction is one of the pathophysiological mechanisms leading to impaired cardiac function.

**Methods:** An adequate animal model for studying pathophysiology of T2DM is the New Zealand Obese (NZO) mouse. These mice were maintained on a high-fat diet (HFD) without carbohydrates for 13 weeks followed by 4 week HFD with carbohydrates. NZO mice developed severe obesity and only male mice developed manifest T2DM. We determined cardiac phenotypes and mitochondrial function as well as cardiomyocyte signaling in this model.

**Results:** The development of an obese phenotype and T2DM in male mice was accompanied by an impaired systolic function as judged by echocardiography and MyH6/7 expression. Moreover, the mitochondrial function only in male NZO hearts was significantly reduced and ERK1/2 and AMPK protein levels were altered.

**Conclusions:** This is the first report demonstrating that the cardiac phenotype in male diabetic NZO mice is associated with impaired cardiac energy function and signaling events.

## Introduction

### Diabetes as Cardiovascular Risk Factor

Obesity and its related diseases, such as type 2 diabetes mellitus (T2DM) and cardiovascular diseases (CVD), developed through increasing caloric intake and/or reduced energy expenditure, are a present critical global health problem. The number of T2DM patients is expanding every year (1). Diabetes is characterized by high blood glucose, either because the body cannot produce enough insulin or is unable to use it effectively, consequently leading to insulin resistance or deficiency (2). It has been shown that T2DM is more common in men than in women (3). Moreover, men develop diabetes at a lower body mass index and are predestined to be more insulin-resistant (4). A recent review by Harreiter and Kautzky-Willer pointed out the importance of the investigation of sex differences in prevention of T2DM (5). The authors addressed that trials investigating lifestyle or pharmacological intervention in males and females at risk were promising so far, but that there is more need to analyze biological and psychosocial differences among women and men. In addition, T2DM increases the risk of CVD, known to be a major cause of morbidity and mortality in diabetic men and women (6–8).

### Sex Differences in Heart Metabolism

Interestingly, marked sex differences of healthy individuals regarding mitochondrial function in the heart have been reported. In fact, female rat heart mitochondria produce less reactive oxygen species and had a greater antioxidant capacity than those from males (9), resulting in a better protection of heart function in females (10). Cardiac metabolic response is regulated by several intracellular signaling pathways, including the mitogen activated protein kinase (MAPK) signaling. One class of the MAPKs is the extracellular signal regulated kinases 1/2 (ERK1/2), mediating heart development, metabolism and function. Cumulative evidence confirms that MAPKs influence cardiac compensation and decompensation partly through mitochondria interactions (11). Previous MAPKs studies demonstrated a direct interaction with the outer mitochondrial membrane, translocation into mitochondria (12–14) and indirect effects between these kinases and mitochondria (15–20). Additionally, a key player in the modulation of metabolism is AMP-activated protein kinase (AMPK) (21). Recently, AMPK was shown to be required for the fragmentation of mitochondria and is sufficient to induce mitochondrial fission (22). Two other very important functionally distinct cardiac proteins, responsible for contractility, are the myosin heavy chain isoforms alpha (Myh6) and beta (Myh7). Relative expression levels are altered in cardiac disease (23). However, there is a gap regarding our knowledge on sex dimorphism in diabetes associated cardiovascular research (24). In addition, most preclinical research is done using male animals or cells with undefined/unmentioned sex (25, 26).

### The New Zealand Obese Mouse as a Model for Diabetes

The New Zealand Obese (NZO) mouse is an appropriate animal model to examine sex differences in diabetes and related cardiac function. The NZO mouse represents a model of morbid obesity, insulin resistance, hypertension, and hypercholesterolemia which resembles the human metabolic syndrome (27). Obesity in the NZO mouse is the consequence of a moderately increased food intake and reduced energy expenditure. It is accompanied by a marked hyperglycemia and hyperinsulinemia at earlier age, followed by associated beta-cell destruction (27). Phenotypically, overt diabetes in NZO mice is defined by a threshold of 16.6 mM plasma glucose (27). Interestingly, NZO mice possess sex-dependent characteristics. While female and male NZO mice become obese on a high-fat diet, females are protected from becoming diabetic. In males instead, the diabetes prevalence is usually 50–75% at the age of 22 weeks (28, 29). However, it has been shown that female NZO mice can develop diabetes on a high-fat-diet (HFD) (30). Surprisingly, cardiovascular parameters considering heart functions in the NZO mice have not yet been investigated in relation to metabolic hear function.

In this study, we investigated whether changes in the diabetic state are associated with sex, and this correlates with changes in cardiac mitochondrial function or expression of metabolically-associated proteins and contractile proteins and cardiac function in the NZO mouse.

## Materials and Methods

### Experimental Design

Mice were kept in agreement with the National Institutes of Health guidelines for care and use of laboratory animals. All animal procedures were performed in accordance with the guidelines of the German Law on the Protection of Animals and were approved by the local authorities (Landesamt für Gesundheit und Soziales, Berlin, Germany).

Male and female NZO/HIBomDife (German Institute of Human Nutrition Potsdam-Rehbruecke [DIfE], Nuthetal, Germany) and C57BL/6JRj (B6, control group) mice from Janvier were housed under identical conditions (12 light/dark cycle, 21°C room temperature and free access to food and water). The number of animals is indicated in the legends of the figures. At 5 weeks of age, mice were placed on a HFD with a low carbohydrate content (Altromin, custom made by the manufacturer, Lage, Germany: C 1057-89; fat: 30.5%, protein: 32%, carbohydrates: <0.1%) and at an age of 18 weeks, the animals obtained a high carbohydrate diet (HCD) (Altromin, custom made by the manufacturer: C 1090-60; fat: 35%, protein: 21.4%, carbohydrates: 29%) for 4 weeks. Body weight and blood glucose levels were weekly controlled. Body weight was measured with an electronic scale and blood glucose was determined with a Contour XT glucose meter (Bayer Health Care, Leverkusen, Germany). Blood samples were collected before sacrificing the mice and were stored after centrifugation at −80°C. For the oxygen consumption experiment hearts were cut longitudinally and fiber bundles were isolated. The remaining heart tissue was frozen in liquid nitrogen and stored at −80°C until protein or mRNA isolation.

### Serum Insulin- and Proinsulin-ELISA

The concentration of insulin and proinsulin in murine serum was determined by using the Mouse High Range Insulin ELISA (ALPCO, Salem, USA) and carried out according to the manufacturer's instructions.

### Mitochondrial Respiration Function

The mitochondrial function of heart fibers was measured by oxygraph chambers and Clarke electrodes from Hansatech. A heart-fiber bundle was removed and fibrillated to form individual fibers that were still connected at the ends. These fibers were permeabilized under stirring for 30 min in saponin buffer and then washed twice for 10 min. From the permeabilized fibers 2 × 2 mm pieces (about 3–5 mg wet weight) were separated and placed in 1 ml potassium chloride buffer in the oxygraph chambers. The chambers were closed airtight and the basal respiration of the fibers was determined. Thereafter, the oxygen consumption was recorded and presented as a graph during adding the following substrates and metabolites in an interval of about 2 min: 2.5 mM adenosine diphosphate (ADP), 4 μM rotenone, 10 mM succinate, 8 μM cytochrome c, 4 μM Antimycin A, 0.5 mM tetramethyl-p-phenylenediamine (TMPD), and 7.5 mM sodium azide. At the end, the respiratory control index (RCI), the ratio of maximum respiration (state 3) and respiration in the absence of ADP (state 4), was determined by the software program.

### RNA and Protein Analysis

The mRNA isolation from 5 mg heart tissue was done with the Dynabeads™ mRNA Purification Kit (Thermo Fisher Scientific # 61006). According to the manufacturer's instructions mRNA samples were reverse transcribed with the SensiFAST cDNA Synthesis Kit (Bioline # BIO-65053) and quantitative real time-PCR reactions in the presence of Dream-Taq-Hot Start- DNA Polymerase (Thermo Fisher Scientific # EP1703) and SYBR Green (Life Sciences) was performed. Following murine primers were used: myosine heavy chain 6 (Myh6) (fwd 5′-AGAAGCCCAGCGCTCCCTCA-3′, rev 5′- TGCCTCGGGTCAGCTGGGAA-3′, myosine heavy chain 7 (Myh7) (fwd 5′-TTCCTTACTTGCTACCCTC-3′, rev 5′-CTTCTCAGACTTCCGCAG-3′). Relative abundance of mRNA was calculated after normalization to ribosomal protein L13a (Rpl13a) reference (primer sequence: fwd 5′-GTTCGGCTGAAGCCTACCAG-3′, rev 5′-TTCCGTAACCTCAAGATCTGCT-3′.

Protein isolation of approximately 10 mg of tissue was performed in 500 μl of RIPA buffer (including protease inhibitors) using ceramic spheres in a homogenizer (FastPrep-24, MP Biomedicals). The concentration of the protein samples was determined by a BCA Protein Assay Kit (Pierce # 23225). Lysates were analyzed by immunoblotting using primary antibodies raised against phosphor-p44/42 MAPK (pERK) (Cell Signaling #4370), p44/42 MAPK (ERK1/2) (Cell Signaling #4695), phospho-AMPKα (Thr172) (Cell Signaling #2535), AMPKα (Cell Signaling #2532), Myh6 (K-13) (Santa Cruz #168676), Myh7 (Santa Cruz #71575), α-tubulin (Sigma #T9026), and secondary antibodies, such as donkey anti-mouse IgG, donkey anti-rabbit-IgG or donkey anti-goat-IgG antibodies (Jackson Immuno Research Laboratories). For the investigation of mitochondrial respiration chain complexes a total OXPHOS Rodent WB Antibody Cocktail (abcam #110413) was taken. For detection, enhanced chemiluminescent reagents (ECL kit; Thermo Scientific) and a BioRad Chemidoc MP System were used.

### Echocardiography

Echocardiographic analysis regarding cardiac morphology and function was carried out at the age of 22 weeks. Echocardiography was performed as previously described, with the exception that the isofluran dose needed to be adapted to the higher weight of animals (3–4%) (31, 32). Echocardiography was performed using a MX400 ultra-high frequency linear array transducer (18–38 MHz, center transmit: 30 MHz, axial resolution: 50 μm) together with a Vevo® 3100 high-resolution Imaging System (both FUJIFILM VisualSonics, Toronto, ON, Canada). M-Mode images of the maximum dimension of the LV in parasternal long axis view were used to analyze cardiac dimensions and calculate LV mass (LVM). B-Mode pictures were obtained in order to analyze cardiac volumes and LV function parameters. All data sets were acquired prospectively and analyzed for this study in a retrospective manner.

### Statistics

All analyses were performed using GraphPad Prism 7. Results represent mean values ± standard deviation (SD). Unpaired *t*-test or Mann-Whitney test and for comparison of the multiple groups, two-way ANOVA followed by Tukey-B-Posthoc-test was used. Differences with *p* ≤ 0.05 were considered statistically significant.

## Results

### Manifestation of Obesity and Diabetes

**Figure 1:**
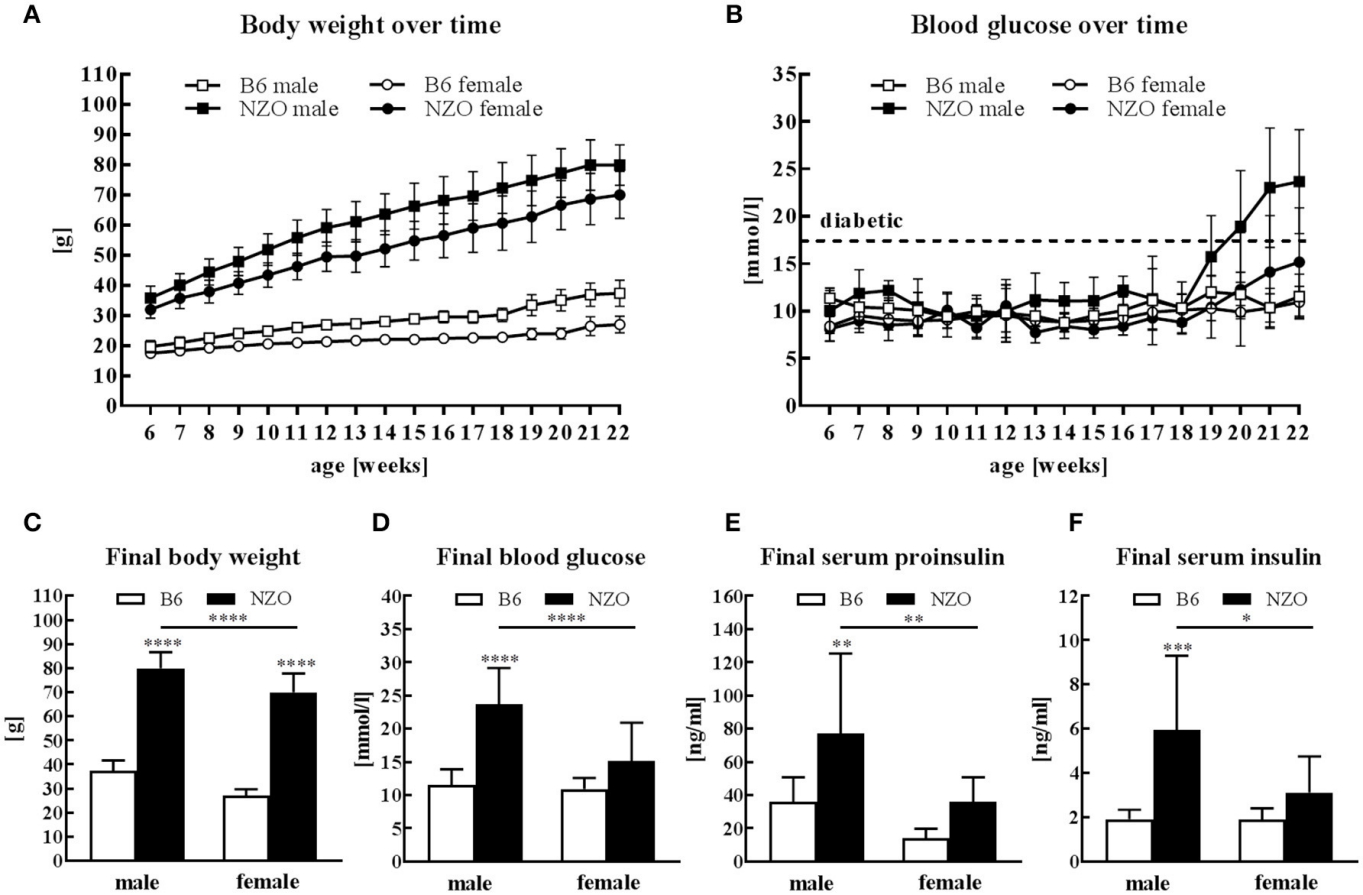
Body weight, glucose and insulin in male and female NZO and B6 mice. Body weight **(A)** and blood glucose **(B)** course during the whole study. Body weight **(C)**, blood glucose **(D)**, serum proinsulin **(E)** and serum insulin **(F)** at the end of the study (week 22). Mean ± SD. Two-way ANOVA with Tukey's posthoc test, ^*^p < 0.05, ^**^p < 0.01, ^***^p < 0.001, ^****^p < 0.0001; B6 male, *n* = 10–12; NZO male, *n* = 10–18; B6 female, *n* = 10–12; NZO female, *n* = 10–19.

All mice received HFD without carbohydrates for 13 weeks followed by 4 weeks HCD. Male and female NZO mice developed severe obesity until the end of the study compared to the B6-control group (Figure 1A). Blood glucose levels of NZO males and females were also increased in comparison to B6 mice, but only if mice were treated for 4 weeks with HCD (Figure 1B). Comparing the final body weight of male and female NZO with the corresponding B6 at 22 weeks, body weight of both sexes was significantly increased (Figure 1C). However, exclusively the male NZO mice showed significantly higher blood glucose levels compared to their B6 counterparts (Figure 1D). Development of T2DM in male NZO mice was accompanied by significant higher serum proinsulin (Figure 1E) and serum insulin levels compared to B6 controls, while values of female NZO mice did not reach statistical significance (Figure 1F).

### Characterization of Cardiac Phenotype by Echocardiography

Echocardiography was used to phenotype the cardiac performance of HCD-fed NZO mice and B6 controls. First, we determined the cardiac phenotype, i.e., heart rate, LV wall thicknesses, and LVM, and measures of systolic cardiac performance (Table 1, first part). NZO males had a dramatically lowered heart rate when compared to B6 control males. The LV anterior and posterior walls were significantly thicker in NZO males than in B6 males. In female NZO mice exclusively the LV posterior wall demonstrated moderately increased wall thickening compared to female B6 mice. The LVM was significantly increased in both NZO sexes in comparison to corresponding B6 mice, and was also significantly increased in NZO males when compared to NZO females, which was not the case when looking at B6 males compared to B6 females. The inner diameters of the LV were significantly increased in both NZO sexes in comparison to corresponding B6 mice. No differences were observed, when comparing ESV and EDV of NZO mice with their sex-matched B6-controls. NZO mice of both sexes tended to have greater ESVs but smaller EDVs compared to sex-matched B6 controls, however reaching no statistical significance (Table 1, second part). In both genotypes, female mice showed lower absolute ESV and EDV compared to corresponding male controls. SV and EF were significantly decreased in both sexes of NZO mice when compared to sex-matched B6 controls.

**Table 1:** Echocardiographic-assessed characterization of cardiac phenotypes after 22 weeks.

	**B6 male**	**NZO male**	**B6 female**	**NZO female**
**PHENOTYPE**				
n-number	12	18	12	19
Heart rate, bmp	528.1 ± 45.3	450.9 ± 83.1^**^	494.4 ± 37.7	495.4 ± 56.9
LVAW, d, mm	0.67 ± 0.09	0.75 ± 0.07^*^	0.66 ± 0.09	0.70 ± 0.07
LVPW, d, mm	0.60 ± 0.03	0.79 ± 0.07^****^	0.55 ± 0.08	0.73 ± 0.08^****^
LVID, d, mm	2.77 ± 0.26	4.20 ± 0.27^****^	2.52 ± 0.21	3.95 ± 0.32^****^
LVM, mg	68.98 ± 11.18	96.88 ± 14.87^****^	55.93 ± 11.67	78.81 ± 11.04^****,###^
**FUNCTION**				
ESV, μl	28.96 ± 7.40	32.78 ± 7.73	22.6 ± 3.44	25.88 ± 9.06^#^
EDV, μl	75.10 ± 17.63	67.35 ± 10.62	59.77 ± 8.55	53.72 ± 8.15^##^
SV, μl	46.13 ± 11.00	34.57 ± 7.63^***^	37.18 ± 6.10	27.84 ± 5.15^**,#^
EF, %	61.46 ± 3.42	51.29 ± 8.60^**^	62.07 ± 3.52	52.82 ± 11.36^*^

Mean ± SD, Two-way ANOVA with Tukey's posthoc test, ^*^p < 0.05, ^**^p < 0.01, ^***^p < 0.001, ^****^p < 0.0001. ^*^, B6 vs. NZO; ^#^, NZO male vs. NZO female; LVAW, d, left ventricular anterior wall (diastole); LVPW, d, left ventricular posterior wall (diastole); LVID, d, left ventricular inner diameter (diastole); LVM, left ventricular mass; BW, body weight; FS, fractional shortening; EDV, end-diastolic volume; ESV, end-systolic volume; SV, stroke volume; EF, ejection fraction.

### Changes of Cardiac Contractile Proteins

**Figure 2:**
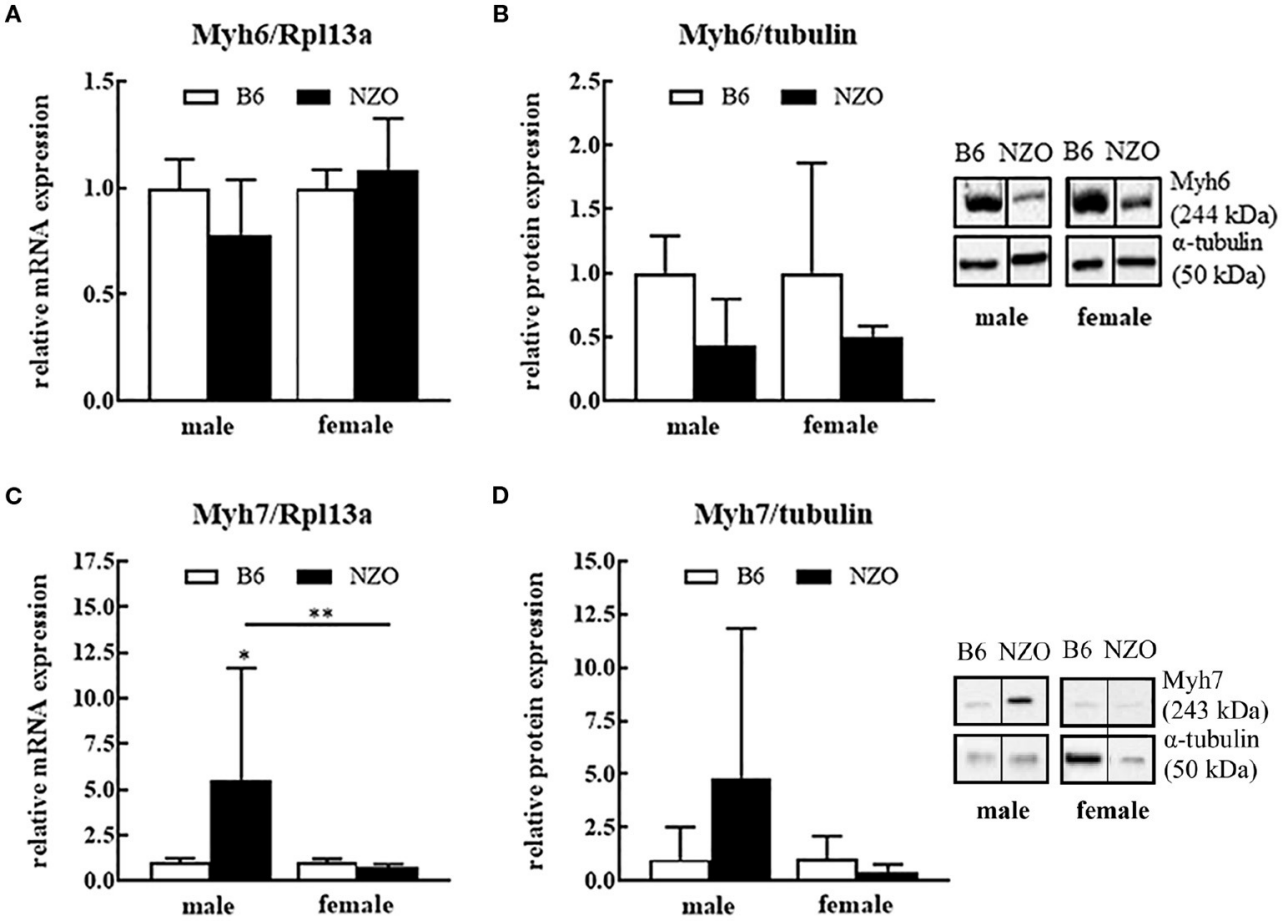
Protein and mRNA expression differences of cardiac myosine heavy chain isoforms 6 and 7. Myh6 mRNA expression level **(A)**, Myh6 protein expression **(B)**, Myh7 mRNA expression **(C)** and Myh7 protein expression **(D)**. mRNA was normalized to Rpl13a and as protein expression reference α-tubulin was used. Mean ± SD. Two-way ANOVA with Tukey's posthoc test, ^*^p < 0.05, ^**^p < 0.01; B6 male: *n* = 5–10, NZO male: *n* = 7–10, B6 female: *n* = 5–10, NZO female: *n* = 7–10.

Next, we investigated expression levels of contractile proteins Myh6 and Myh7. Male NZO mice showed a tendency to a lower Myh6 mRNA and protein expression level compared to B6 males. Female NZO mice had no changes in the Myh6 mRNA expression, while theMyh6 protein levels also have a tendency to be lower compared to B6 females (Figures 2A,B). In contrast, in male NZO mice, the Myh7 mRNA expression was significantly increased compared to female NZO and male B6 mice (Figures 2C,D). However, Myh7 mRNA and protein levels were not elevated in female NZO mice, compared to the B6 controls (Figures 2C,D).

### Mitochondrial Function

**Figure 3:**
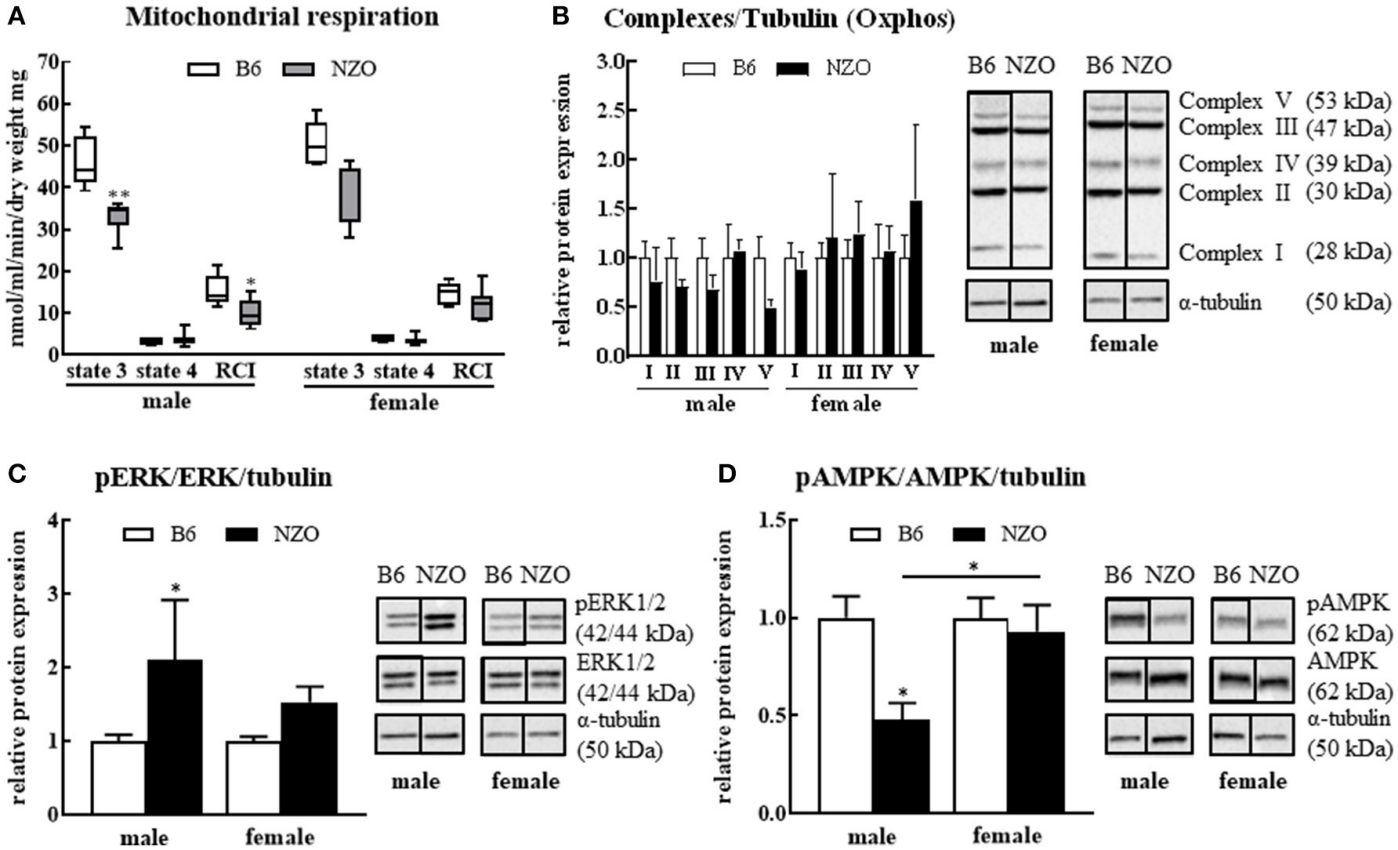
Changes in mitochondrial function and signaling pathways. Mitochondrial respiration **(A)** and protein levels of mitochondrial respiration chain complexes **(B)**, phosphorylated ERK1/2 **(C)** and phosphorylated AMPK **(D)**. α-tubulin was used as reference protein. Mean ± SD. Two-way ANOVA with Tukey's posthoc test, ^*^p < 0.05, ^**^p < 0.01; B6 male: *n* = 5, NZO male: *n* = 7, B6 female: *n* = 5, NZO female: *n* = 7.

Since the major goal of our study was to investigate the influence of a sex-related diabetic phenotype on the energy function of the heart, we next investigated whether changes in mitochondrial function or expression of metabolism-associated proteins in the heart explain the observed sex differences in cardiac phenotype. After sacrificing the mice, single heart fibers were isolated and mitochondrial function was measured. The RCI was significantly reduced in NZO males vs. B6 controls. State 3 was significantly decreased, whereas state 4 showed no changes in male NZO mice compared to B6 males. In contrast to male NZOs, the mitochondrial respiration of female NZO mice was not significantly changed in comparison to B6 mice (Figure 3A). Furthermore, the relative protein expression of all mitochondrial respiration chain complexes, excluding complex IV, seemed to be reduced in NZO males vs. B6 males, although no significant differences were found. In female NZO mice no protein expression change of the complexes was seen (Figure 3B). In general, the protein expression levels of most mitochondrial complexes had a tendency to be higher in females compared to the corresponding males.

In addition, the amount of the phosphorylated ERK1/2 was significantly elevated in male NZO mice compared to male B6 animals (Figure 3C). Inversely, the phosphorylated AMPK protein expression was significantly lower in male NZO mice than in B6 male controls and as well in comparison to female NZO mice (Figure 3D). No significant changes were seen in female NZO mice regarding the protein expression levels of phosphorylated ERK1/2 or AMPK (Figures 3C,D).

## Discussion

Here we were able to show that the development of an diabetic phenotype in male NZO mice was accompanied by changes in the heart function, as judged by the echocardiography, by changes in the Myh6/7 expression and moreover by impairment of mitochondrial function. A reduced state 3 respiration and RCI was accompanied by significantly reduced ERK1/2 and AMPK protein levels.

Male and female NZO mice developed a severe obesity when they were maintained on a HFD until the age of 22 weeks which is in agreement with the study of Kluge et al. (33). After the feeding of carbohydrates from the 18th week of age only male NZO mice revealed a manifest T2DM status whereas female NZO mice seem to be protected from T2DM. Male NZO mice generated a marked hyperglycemia and hyperinsulinemia reflecting pronounced insulin resistance which is consistent with data from Joost and Schürmann (27). In females, the final blood glucose and the associated serum proinsulin as well as serum insulin levels were significantly lower than in male NZO mice and there were no changes to the B6-control group which indicates no overt diabetes development (Figure 1). Interestingly, a previous study showed that an ovariectomy of NZO females modulates diabetic insulin resistance and produced a phenotype which was comparable with that of NZO males (34).

Nevertheless, until now it is not known how this effect influences the onset and progression of cardiovascular events. To our knowledge, we describe here for the first time the cardiovascular damage events in relation to metabolic functions in the NZO mouse model in a sex-sensitive manner. In general a decrease in insulin sensitivity leads to less glucose uptake which perhaps can exert feedback on a molecular regulation leading to an increase in mitochondrial dysfunction in the heart (35). Today, no extensive knowledge exists about the energy metabolism in the diabetic human heart, but it is known that the energy supply through glucose in diabetic hearts is disturbed (36). The influence of sex regarding glucose utilization in obese and diabetic patients seems to be pronounced (37). The exact underlying mechanisms, impairing cardiac function and promoting cardiomyocyte injury, have not yet been demonstrated. Besides lipotoxicity, oxidative stress, intramyocardial inflammation and altered insulin and calcium signaling, mitochondrial dysfunction plays an important role (38). Moreover, hyperlipidemia has been shown to increase stress on mitochondria as they attempt to generate sufficient ATP, leading to increased ROS production (39). In fact, all this leads to further complications in the diabetic heart (40, 41). In our study, the mitochondrial respiration, represented by RCI, was significantly lower in male NZOs than in females where no significant changes were documented compared to the control group. One explanation for the decreased mitochondrial respiration could be that the mitochondrial respiration chain complexes are less expressed in the male NZO hearts (Figure 3, part one). Decreased expression level of mitochondrial chain complexes could either be due to a reduced number of mitochondria per cardiomyocyte or a low expression level of the proteins per mitochondrial unit. We could not study mitochondrial numbers but found a trend toward a lower expression of respiratory proteins in the male NZOs. Interestingly, investigations in other obesity mouse models related to T2DM, such as the ob/ob and the db/db mice, showed likewise decreased cardiac mitochondrial respiration capacity and lower expression of respiration chain complexes (42, 43).

Mitochondrial dysfunction activates various signaling pathways so we tested the phosphorylation level of ERK1/2 and AMPK two markers of mitochondrial metabolism. In our study, the level of pERK was significantly upregulated only in male NZO mice, but there were no differences in female NZO mice vs. the control group (Figure 3C). The activation of ERK1/2 was also shown in other studies with diabetic hearts (44) and it is also known that activated ERK1/2 contributes to apoptosis in cardiomyocytes under diabetic conditions (45, 46). In addition, it was reported that mitochondrial dysfunction was associated with increased activation and protein expression of ERK1/2 and an attenuated mitochondrial respiration and, therefore, ATP production, wherein a decreased complex I activity and substrate oxidation was observed as well (47, 48). A recent study with diabetic db/db mice showed that levels of phosphorylated ERK1/2 were increased and an inhibition of ERK1/2 can prevent cell death under diabetic conditions. The authors concluded a pathological cardiac change in diabetes through ERK1/2 (49). Therefore, these findings are a clear indication of a metabolic failure and stress in diabetic male hearts.

In contrast, pAMPK was significantly decreased in male NZO mice compared to the B6 controls and NZO females. AMPK has been identified in the past as a central actor of mitochondrial homeostasis. The cardiac AMPK downregulation has been reported in some T2DM animal models as an important intervention target (50, 51). Our data are supported by a study in db/db mice in which a significant decrease of AMPK phosphorylation in murine cardiac tissue was observed (52), but oppositely in ob/ob mice an increased cardiac AMPK phosphorylation was observed (53). If this cardiac benefit is secondary to systemic improvement or to intrinsic cardiomyocyte mechanisms remains to be clarified. Impaired cardiac glucose uptake and utilization are evident in T2DM male patients but not in females (54). Moreover, Boudina et al. conclude that the reduced mitochondrial oxidative capacity may contribute to cardiac dysfunction in their obese mouse model (42, 43).

In the present study, we observed as well differences of contractile proteins on sex-specific cardiac phenotypes. Two important heart contractile proteins, Myh6 and Myh7 were altered in the NZO mouse model (Figure 2). While the RNA- and protein expression of Myh6 was decreased in the NZO males, the Myh7 increased, demonstrating clear shift of these two contraction actors. In the non-diabetic female NZO mice there was no change or shift seen. Interestingly, in impaired adult mouse hearts, a shift from the predominant Myh6 toward Myh7 is often present (55). A study from Krenz et al., using transgenic mice, which predominantly expressed Myh7 instead of Myh6 in the heart, showed a significantly reduced cardiac contractility (56). Further, in cardiac hypertrophy an elevation of Myh7 can serve as an early and sensitive marker and recently downregulation of Myh6 expression in human hearts was observed, too (57–59). Transcriptional reprogramming of MHC gene expression has been described to be characteristic for the development of hypertrophy-induced heart failure. Hang et al. have well described that resulting cardiac stress triggers adult or stressed hearts to undergo a shift from α-MHC (Myh6) to β-MHC expression (Myh7) by reactivation of chromatin remodeling protein Brg1. Complexation of Brg1 with its partners PARP and HDAC induce the pathological Myh6 to Myh7 switch (60), that we could also show for the NZO males.

This suggests a cardiac dysfunction in male NZO mice (61–63), which was further verified by echocardiography. Indeed, we observed a lower heart rate in NZO male mice in comparison to the control group, but no changes in female ones. In addition, male NZO mice had significant decreased EF and dramatically increased LVM, indicating more severe cardiac dysfunction than in females. Surprisingly, although in female NZO mice, only developing obesity, not manifest diabetes, we observed a significantly reduced EF and enhanced LVM in comparison with B6 controls, corresponding to a non-significant trend for a decrease in mitochondrial function (Table 1). Nevertheless, it seems that the diabetic status of the male NZO mice aggravated the cardiac dysfunction in males. Males had a lower mitochondrial respiration, increased ERK1/2 and decreased AMPK activity as well as an increased Myh7 expression level in the heart, which was not seen in female NZO mice.

In our NZO diabetic animal model, the cardiac function was severely decreased, particularly in the male NZO mice. It is possible that sex differences in mitochondrial function potentially contribute to a better cardiac performance in females. Thus, from T2DM protected female NZO mice show a better mitochondrial respiration rate than diabetic male NZO mice. Sex-specific changes in the related mitochondrial function and cellular stress signaling let us propose a functional impairment of the heart, which was confirmed by echocardiography.

## Author Contributions

CJ: performed most of the animal treatment, analyzed the data and wrote the manuscript; JG: executed echocardiographic data acquisition and –analysis; CO and KN: helped to prepare the animal tissues; SD: conducted the analysis to serum proinsulin- and insulin levels and RT-PCR; AK: determined the mitochondrial respiration assay and western blots; CS: analyzed the data and proofed the statistics; CO, UK, VR-Z, and TG: designed and supervised the project and enabled the experiments. All authors reviewed the manuscript.

### Conflict of Interest Statement

The authors declare that the research was conducted in the absence of any commercial or financial relationships that could be construed as a potential conflict of interest.
