# Correction: Sex differences in cardiac mitochondria in the New Zealand obese mouse

**DOI:** 10.3389/fendo.2025.1684950

**Published:** 2025-10-09

**Authors:** Cathleen John, Jana Grune, Christiane Ott, Kerstin Nowotny, Stefanie Deubel, Arne Kühne, Carola Schubert, Ulrich Kintscher, Vera Regitz-Zagrosek, Tilman Grune

**Affiliations:** ^1^ Department of Molecular Toxicology, German Institute of Human Nutrition Potsdam-Rehbruecke, Potsdam, Germany; ^2^ German Center for Cardiovascular Research (DZHK), Partner Site Berlin, Berlin, Germany; ^3^ Institute of Physiology, Charité Universitätsmedizin, Berlin, Berlin, Germany; ^4^ Institute of Pharmacology, Center for Cardiovascular Research, Charité -Universitätsmedizin Berlin, Berlin, Germany; ^5^ Center for Cardiovascular Research, Charité Universitätsmedizin Berlin, Berlin, Germany; ^6^ Institute for Gender in Medicine, Charité Universitätsmedizin Berlin, Berlin, Germany; ^7^ Institute of Nutritional Science, University of Potsdam, Potsdam, Germany; ^8^ German Center for Diabetes Research, Oberschleißheim, Germany

**Keywords:** NZO, heart, obesity, mitochondrial function, echocardiography, systolic function

There was a mistake in **Figure 2D** as published. Accidentally, the immunoblot band of **Figure 2B** (male, NZO) was inserted in **Figure 2D** (female, NZO). The corrected Figure appears below.

**Figure 2:**
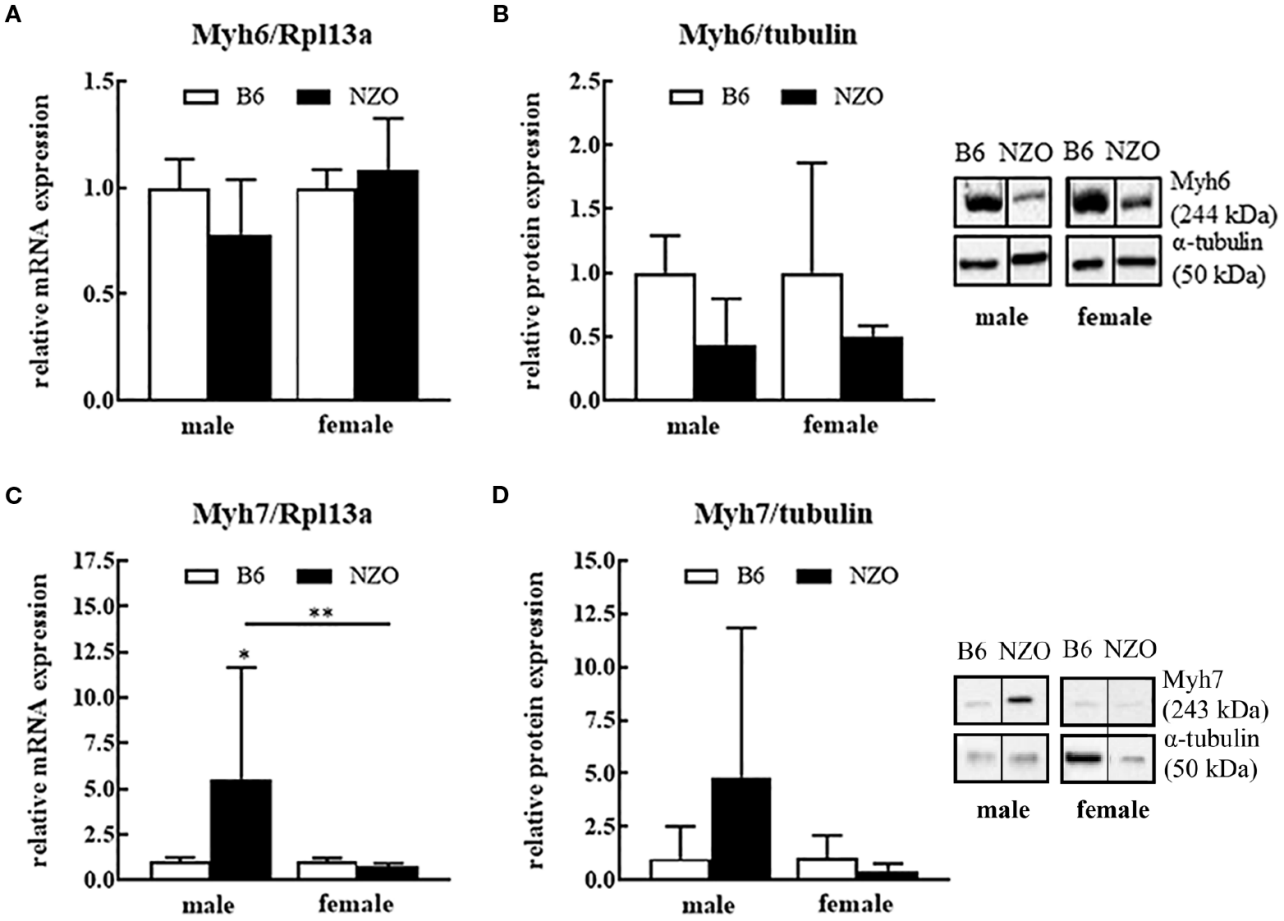
Protein and mRNA expression differences of cardiac myosine heavy chain isoforms 6 and 7. Myh6 mRNA expression level **(A)**, Myh6 protein expression **(B)**, Myh7 mRNA expression **(C)** and Myh7 protein expression **(D)**. mRNA was normalized to Rpl13a and as protein expression reference α-tubulin was used. Mean ± SD. Two-way ANOVA with Tukey's posthoc test, *p < 0.05, **p < 0.01; B6 male: *n* = 5–10, NZO male: *n* = 7–10, B6 female: *n* = 5–10, NZO female: *n* = 7–10.

The original version of this article has been updated

